# The Regulatory Role and Mechanism of Myoferlin in Mitophagy During Papillary Thyroid Carcinogenesis

**DOI:** 10.1002/kjm2.70245

**Published:** 2026-06-10

**Authors:** Wen‐Chao Lyu, Yang‐Shuai Wang, Chun‐Yang Shang, Ming Qi, Tong‐Chang Li, Li‐Xin Lian, Hai‐Quan Qiao

**Affiliations:** ^1^ Department of Gastrosplenic Surgery The First Affiliated Hospital of Harbin Medical University Harbin Heilongjiang Province China; ^2^ Department of General Surgery The First Hospital of Harbin Harbin Heilongjiang Province China

**Keywords:** mitochondrial dysfunction, mitophagy, myoferlin, papillary thyroid carcinoma, PINK1‐Parkin pathway

## Abstract

Papillary thyroid carcinoma (PTC) is the most prevalent subtype of thyroid cancer; however, the regulatory mechanisms by which mitophagy influences its progression remain inadequately elucidated. This study sought to examine the role of myoferlin (MYOF) in mitophagy and its molecular basis during PTC development. Utilizing three paired PTC and adjacent normal tissues, we observed elevated MYOF expression at both protein and mRNA levels through western blot and qRT‐PCR analyses. Stable MYOF knockdown cell lines were established in PTC (TPC‐1, KTC‐1) and normal thyroid (Nthy‐ori 3‐1) cells using lentiviral shRNA. Functional assays, including CCK‐8, wound healing, transwell, flow cytometry, immunofluorescence, and mitophagic flux analysis, along with a xenograft mouse model, were employed. Subsequent evaluations involved hematoxylin and eosin staining, immunohistochemistry, western blot, and qRT‐PCR. As a result, MYOF was significantly upregulated in PTC tissues and TPC‐1 cells. Knockdown of MYOF inhibited PTC cell proliferation, invasion, migration, and colony formation, while promoting apoptosis. Mechanistically, MYOF was found to regulate PTC progression through the PINK1/Parkin‐mediated mitophagy pathway. In vivo xenograft experiments demonstrated that MYOF silencing suppressed tumor growth and increased the expression of mitophagy‐related proteins BNIP3 and NIX. In conclusion, MYOF drives PTC progression by repressing mitophagy. Targeted inhibition of MYOF activates tumor‐suppressive mitophagy via the PINK1‐Parkin axis, indicating MYOF as a potential therapeutic target in PTC.

## 1 Introduction

Thyroid cancer (TC) represents the most prevalent head and neck cancer with a rapidly increasing global incidence (6.2% annual growth) [1]. Currently ranking as the fifth most common malignancy in women [2], the histopathological spectrum of TC is dominated by papillary thyroid carcinoma (PTC)—accounting for > 80% of cases and exhibiting the most rapid increase among solid tumors [3, 4]. Despite generally favorable outcomes, 15%–30% of patients with PTC develop locoregional recurrence or lymph node metastasis [5]. Current targeted therapies (e.g., BRAF) demonstrate limited efficacy due to acquired resistance, which fail to fundamentally alter disease progression. Thus, identifying resistance mechanisms and developing novel therapeutic strategies constitute critical unmet needs.

Mitophagy represents a selective autophagic process that eliminates dysfunctional mitochondria, thereby preserving mitochondrial integrity and cellular homeostasis [6]. Defective mitophagy drives metabolic reprogramming toward glycolysis, which reduces the half‐maximal inhibitory concentration (IC_50_) and positions it as a therapeutic target [7]. Concurrently, tumor‐derived exosomes transfer mutated mitochondrial DNA (mtDNA) to CD8^+^ T cells, which suppress T cell mitophagy and facilitates immune evasion [8, 9]. In PTC, autophagy promotes epithelial–mesenchymal transition (EMT) through E‐cadherin degradation and transcription factor release, which develops an autophagy‐EMT‐ferroptosis positive feedback loop that improves metastatic potential [10]. These mechanisms collectively indicate mitophagy as a pivotal regulator in cancer pathogenesis, highlighting its therapeutic relevance for PTC intervention.

Myoferlin (MYOF), a dysferlin‐family membrane protein [11], orchestrates vesicular trafficking and plasma membrane repair. Its overexpression correlates with oncogenic progression across malignancies, thereby mediating invasive protrusion formation and membrane injury response [11, 12]. MYOF depletion attenuates migration/invasion capacities in breast cancer models, while mechanistically improving fatty acid uptake via CD36 stabilization to sustain β‐oxidation [13, 14, 15]. Notably, MYOF knockout induces adenosine triphosphate (ATP)/high mobility group box‐1 protein (HMGB1) release—activating CD8^+^ T cell infiltration [16]. This immunomodulatory mechanism parallels mitophagy enhancement by mitochondrial‐targeted nano‐chimeras, indicating conserved metabolic‐immune crosstalk. Integrated findings reveal a close association between MYOF and mitophagy.

By employing multidimensional analytical approaches, this study delineates the multifaceted regulatory role of MYOF in mitophagy, which defines the precise molecular mechanisms underlying its involvement in PTC pathogenesis. The outcomes are expected to help develop a novel theoretical framework for understanding PTC progression and provide innovative avenues for improving clinical outcomes in patients with refractory PTC.

## 2 Materials and Methods

### 2.1 Clinical Sample Collection

Tissue samples (*n* = 3 paired sets), comprising PTC tissues and matched adjacent tissues, were collected from the First Affiliated Hospital of Harbin Medical University on January 17, 2025. The PTC tissues were designated as the experimental group, whereas histologically confirmed tumor‐adjacent normal tissues (resected ≥ 2 cm from the tumor margin) served as the control group. The Ethics Committee of First Affiliated Hospital of Harbin Medical University approved the research protocol (Authorization Code: IRB‐AF/SC‐08/07.0). The study procedures strictly adhered to the Declaration of Helsinki principles, and all enrolled participants signed written informed consent. GraphPad Prism software was used for statistical analyses, with *p*‐values of < 0.05 indicating statistical significance.

### 2.2 Cell Transfection

Lentiviral transduction was performed in TPC‐1 and KTC‐1 cells to generate stable lines that express MYOF‐targeting shRNA or negative control shRNA (NC), thereby developing PTC cellular models (designated MYOF‐shRNA TPC‐1/KTC‐1 and MYOF‐shNC TPC‐1/KTC‐1). Cells were cultured to achieve 80%–95% confluency before experimental procedures. After diluting plasmid DNA (Table 1 sequences) in 1.5 mL of Opti‐MEM, 24 μL of Transfection‐mate reagent was separately diluted in 1.5 mL of Opti‐MEM and incubated at room temperature (RT) for 5 min. The solutions were combined, adjusted to 3 mL with culture medium, vortexed gently, and incubated (20 min, RT). The transfection mixture was added to the dishes after aspirating the culture medium. Cells were maintained at 37°C/5% CO_2_ for 24 h before gene expression assessment.

**TABLE 1:** Sequence information used in this study.

The MYOF interference sequence (5′ to 3′)		
sh‐1:GCTGATCTCCCTGCTAAATGACGAATCATTTAGCAGGGAGAT CAGC		
sh‐2:GCAGGATCTTGGTTGAATTAGCGAACTAATTCAACCAAGATC CTGC		
sh‐3:GGACATCACACCCAAACTTCTCGAAAGAAGTTTGGGTGTGA TGTCC		
Transfection sequence information of si‐PINK1		
si‐PINK1: GGAGAAUGCUGAAGAAUGA		
Primer sequence		
	Forward primer	Reverse primer
MYOF	GCCAACTTCATCCAACACCT	GTGGACAGCTCCTCCTCTTG
PINK1	CGTGGCTTTGGCTGGAGAGTA	CAGGAGGGGCACAGATGAGGT
Parkin	AGCTGCGTGTGATTTTTGCCG	CTGAGGTGCTCTGGGGTTCGT
BNIP3	TCCAGCCTCGGTTTCTATTT	TTGGTATCTTGTGGTGTCTGC
NIX	CTTTGGGGCTGGGCATCTAT	TACACCACTTCACAGGCCAC
BAX	CCTTTTGCTTCAGGGTTTCAT	CGCTTCAGCTTCTTGGTGGAT
Bcl‐2	CGACGACTTCTCCCGCCGCTACCGC	CCGCATGCTGGGGCCGTACAGTTCC
GAPDH	GATTGTTGCCATCAACGACC	GTGCAGGATGCATTGCTGAC

### 2.3 RNA Extraction and Reverse Transcription‐Quantitative Polymerase Chain Reaction (qRT‐PCR)

Total RNA was isolated with TRIzol Reagent (Thermo Fisher Scientific, USA). A ComWin Biotech (China) reverse transcription kit was used to subsequently perform cDNA synthesis under specified conditions (42°C/15 min to 85°C/5 min). SYBR Premix Ex Taq (Yeasen Biotechnology, China) on a Bio‐Rad CFX Connect 96 system (USA) with triplicate technical replicates was employed for qRT‐PCR analyses. The thermal profile comprised: initial DNA denaturation at 95°C for 30 s, followed by 40 amplification cycles, each comprising 10‐s denaturation at 95°C and 30‐s combined annealing/extension at 60°C. GAPDH served as the endogenous control for 2^−ΔΔCt^ normalization.

### 2.4 Western Blot Analysis

Cells/tissues were lysed in radioimmunoprecipitation assay buffer containing 1% phenylmethylsulfonyl fluoride (30 min). After centrifugation (12,000*g*, 4°C, 20 min), supernatants were collected, and protein concentrations were identified employing a commercial bicinchoninic acid assay system (Beyotime Biotechnology, China). Samples were diluted to uniform concentrations, denatured at 100°C for 5 min, and subjected to electrophoresis on 10% SDS‐polyacrylamide gels. Separated proteins were transferred onto polyvinylidene fluoride or polyvinylidene difluoride membranes. After blocking with 5% nonfat milk (RT, 1 h), membranes were incubated overnight at 4°C with the following primary antibodies according to manufacturer‐specified dilutions: MYOF (A15427, ABclonal, Wuhan, China), Parkin (A0968, ABclonal, Wuhan, China), NIX (A24803, ABclonal, Wuhan, China), PINK1 (A7131, ABclonal, Wuhan, China), p‐PINK1 (Proteintech, 23274‐1‐AP, Wuhan, China), BNIP3 (A5683, ABclonal, Wuhan, China), Bcl‐2 (A19693, ABclonal, Wuhan, China), Bax (A19684, ABclonal, Wuhan, China), and GAPDH (M171‐3, BBI, Toronto, ON, Canada). Membranes were subjected to 1 h RT incubation with HRP‐conjugated goat anti‐rabbit/mouse secondary antibodies (1:5000; BBI, Canada). Protein bands were visualized on a BLT Photonics GV6000M2 system (China) and quantified via ImageJ, with expression levels normalized against GAPDH.

### 2.5 Cell Proliferation Assay (CCK‐8)

Log‐phase cells were seeded in 96‐well plates (100 μL/well). At 24, 48, and 72 h postseeding, 10 μL of CCK‐8 solution (Kerme, China) was added following the manufacturer's protocol. After 1‐h dark incubation (37°C, 5% CO_2_), absorbance at 450 nm was recorded using a Biotek microplate reader (USA). Triplicate wells in three independent experiments were analyzed.

### 2.6 Colony Formation Assay

Log‐phase cells were trypsinized, centrifuged, and resuspended to 2 × 10^3^ cells/well. Single‐cell suspensions seeded in 6‐well plates were incubated (37°C, 5% CO_2_) until full attachment (24 h). After initial medium replacement with fresh complete medium (changed every 2–3 days), colonies cultured for 7–14 days underwent phosphate‐buffered saline (PBS) washing (×3), fixation with 4% paraformaldehyde (1 mL/well, 30 min RT), and staining with 1% crystal violet (Biosharp, China; 20 min RT). After PBS removal of excess dye, plates were air‐dried. A digital imaging system (Nikon Eclipse Ti, Tokyo, Japan) was used to document colony formation, with colony formation rates quantified using ImageJ software. Three independent experiments with technical triplicates were conducted.

### 2.7 Flow Cytometry Analysis

Cells in the logarithmic growth phase were trypsinized, seeded into 6‐well plates at optimal density, and allowed to adhere before experimental treatments. An Annexin V‐FITC/PI Kit (Yeasen Biotech, China) was used to assess apoptosis, following the manufacturer's protocol, and analyzed via BD FACSCalibur flow cytometer (BD Biosciences, USA). For reactive oxygen species (ROS) measurement, cells were incubated with 10 μM of 2′‐7′dichlorofluorescin diacetate in serum‐free medium (37°C, 20 min, dark), washed with ice‐cold PBS, and counterstained with 1 μg/mL of 4′,6‐diamidino‐2‐phenylindole (DAPI) (15 min). The BD FACSCanto II cytometer, with 488 nm excitation and 525 nm emission configurations, was used to quantify ROS levels. Meanwhile, for immunofluorescence staining, the cells on glass coverslips were fixed with 4% paraformaldehyde for 15 min at room temperature after PBS washing, permeabilized with 0.1% Triton X‐100 for 10 min and blocked with 5% bovine serum albumin (BSA) for 1 h at room temperature, then counterstained with 1 μg/mL DAPI for 15 min in the dark to label cell nuclei; subsequently, the coverslips were mounted on glass slides with anti‐fluorescence quenching mounting medium, and the intracellular ROS fluorescence signals were observed and captured under a fluorescence microscope. All experiments included three independent biological replicates with technical triplicates.

### 2.8 Cell Migration Assay (Wound Healing)

Log‐phase cells underwent trypsinization, centrifugation, and resuspension before being seeded in 12‐well plates. Cultivation continued until > 95% confluency was achieved. Sterile 10‐μL pipette tips were used to create uniform scratches perpendicular to the plate bottom. Serum‐free medium was applied after PBS washes to remove dislodged cells. An inverted phase‐contrast microscope (Tiannuoxiang, China) was used to document wound closure at 0/24 h. Central scratch regions were photographed at ×40 magnification. Three independent experiments with biological triplicates were conducted. GraphPad Prism 8.0.1 was used to statistically analyze data.

### 2.9 Transwell Migration Assay

Transwell chambers (8 μm) received upper‐surface coating with reduced‐growth‐factor Matrigel (1:8 in serum‐free medium), followed by 37°C polymerization for 3 h. Subsequently, 500 μL of basal medium was added to hydrate the basement membrane for 30 min at 37°C. After removing the liquid, logarithmically growing cells were seeded in the upper chamber at optimal density (2.5 × 10^4^ cells/well). Corresponding chemoattractants were added to the lower chamber. After 24 h of incubation at 37°C with 5% CO_2_, nonmigrated cells on the upper membrane surface were removed using cotton swabs. Migrated cells were fixed using 4% paraformaldehyde for 15 min and stained with 0.1% crystal violet for 20 min. After PBS washing, migrated cells in six stochastic fields per membrane were photographed under an inverted phase‐contrast microscope (×40 objective). Three independent experiments with technical triplicates were performed.

### 2.10 JC‐1 Mitochondrial Membrane Potential Assay

Upon reaching the desired density during the logarithmic (log) phase, cells were harvested, triple‐rinsed with PBS, and stained with JC‐1 working solution (prepared following the JC‐1 Mitochondrial Membrane Potential Assay Kit protocol, Yeasen, Shanghai, China). Staining proceeded for 15 min at 37°C under 5% CO_2_. After incubation, cells were gently washed with PBS to eliminate excess dye. Finally, 2 mL of PBS was added before image acquisition using a Tiannuoxiang (Beijing, China) fluorescence microscope.

### 2.11 Transmission Electron Microscopy (TEM)

After trypsinization, cells from each treatment group were centrifuged (1000 rpm, 5 min). The resulting pellets were harvested and fixed overnight at 4°C using 2.5% glutaraldehyde. After three PBS washes, postfixation was performed with 1% osmium tetroxide for 2 h. Samples were then sequentially dehydrated in graded ethanol solutions, followed by two 15‐min acetone substitution steps. Embedding in resin ensued. Ultrathin sections (70 nm thick) were cut using an ultramicrotome and subsequently double‐stained using uranyl acetate and lead citrate. A FEI Tecnai G2 Spirit transmission electron microscope was used to visualize autophagosome and mitochondrial ultrastructural alterations. Representative images from different fields of view were captured and archived.

### 2.12 Immunofluorescence Staining for Mitochondria and Autophagosomes

After fixation with 4% paraformaldehyde (15 min) and subsequent washing, cells were permeabilized using 0.5% Triton X‐100 for 15 min. Subsequently, blocking was performed with 5% BSA at RT for 30 min. Samples were then incubated overnight at 4°C with primary antibodies against mitochondrial markers (mito) and autophagosome markers (LC3). The following day, after three PBS washes, cells were incubated with fluorescent secondary antibodies (1:500 dilution) at RT for 30 min, protected from light. After additional washes, nuclei were counterstained with DAPI for 5 min under light‐protected conditions. Finally, specimens were mounted in glycerol, and a Leica TCS SP8 laser scanning confocal microscope (Leica Microsystems) was used to acquire images. Fluorescence images of mitochondria (green) and autophagosomes (red) were captured.

### 2.13 Tumor Model Establishment

After acclimatization in SPF barrier facilities (12‐h light/12‐h dark photoperiod with free access to food and water), BALB/c nude mice were implanted subcutaneously in the right axilla with 100 μL suspensions of lentiviral‐transduced stable cells (MYOF‐knockdown or control groups; 5 × 10^7^ cells/mL) under isoflurane anesthesia, with postoperative analgesia using 0.1% butorphanol for 3 consecutive days. Tumor volume (*V* = [*L* × *W* ^2^]/2, *L*: longest diameter, *W*: perpendicular width) and body weight were monitored biweekly until reaching ethical endpoints (maximal tumor diameter = 2 cm), whereupon cervical dislocation was performed for tumor excision and subsequent analysis. The Ethics Committee of Experimental Animal Welfare and the Ethics Committee of Jilin Bairen Medical Laboratory Co. LTD approved the animal experiments (BR.No. 20250306001).

### 2.14 Histological Processing and Staining (H&E)

After 6 h of fixation in 4% paraformaldehyde, tissue specimens were subjected to immobilization comprising ethanol dehydration, xylene clearing, and paraffin embedding. Hematoxylin staining (5 min) preceded acid ethanol differentiation and bluing under running tap water. Subsequent eosin counterstaining was followed by dehydration in ascending ethanol, xylene clearing, and neutral resin mounting. Finally, a light microscope (Motic MF31‐M; Motic, Xiamen, China) was used to examine and image stained sections.

### 2.15 Immunohistochemistry (IHC)

Paraffin‐embedded tissue sections mounted on slides were baked in a 40°C oven for 0.5–2 h. After deparaffinization, tissue sections underwent antigen retrieval treatment, followed by 30‐min blocking with 5% BSA. Appropriately diluted primary antibodies were subsequently applied and incubated at 4°C overnight. The next day, after primary antibody removal, HRP‐conjugated secondary antibodies were subjected to 30‐min exposure at ambient temperature. Diaminobenzidine substrate was applied for 5–10 min for color development. After aqueous rinsing, sections received hematoxylin counterstaining and progressed through graduated ethanol dehydration and xylene clearing, and were then mounted in neutral resin. A Motic MF31‐M light microscope (Motic, Xiamen, China) was used for the final examination and image acquisition of specimens.

### 2.16 Bioinformatic Analysis of MYOF Expression and Clinical Correlations in TCGA‐TC

RNA‐sequencing expression data (TPM) and corresponding clinical information for thyroid carcinoma (TC) patients were downloaded from The Cancer Genome Atlas (TCGA) database (https://portal.gdc.cancer.gov.gov/). The expression level of MYOF was compared between tumor and normal tissues using the Wilcoxon rank‐sum test. Associations between MYOF expression and clinicopathological characteristics, including T stage, N stage, M stage, and pathological grade, were also evaluated using the Wilcoxon rank‐sum test or non‐parametric trend analysis. For survival analysis, univariate Cox regression analysis was performed to assess the prognostic value of MYOF expression. Patients were further divided into high‐ and low‐ MYOF expression groups based on the median expression cutoff. Survival differences between the two groups were compared using the log‐rank test. A *p*‐value < 0.05 was considered statistically significant.

### 2.17 Statistical Analysis

Data are presented as mean ± standard deviation. All experiments were independently repeated at least three times. One‐way analysis of variance with a Bonferroni post hoc test was used for intergroup comparisons. GraphPad Prism version 8.0.1 software was used for statistical analyses, with a *p*‐value of < 0.05 indicating statistical significance.

## 3 Results

### 3.1 Clinical Analysis of MYOF Expression Profiles in PTC Tissues

**FIGURE 1:**
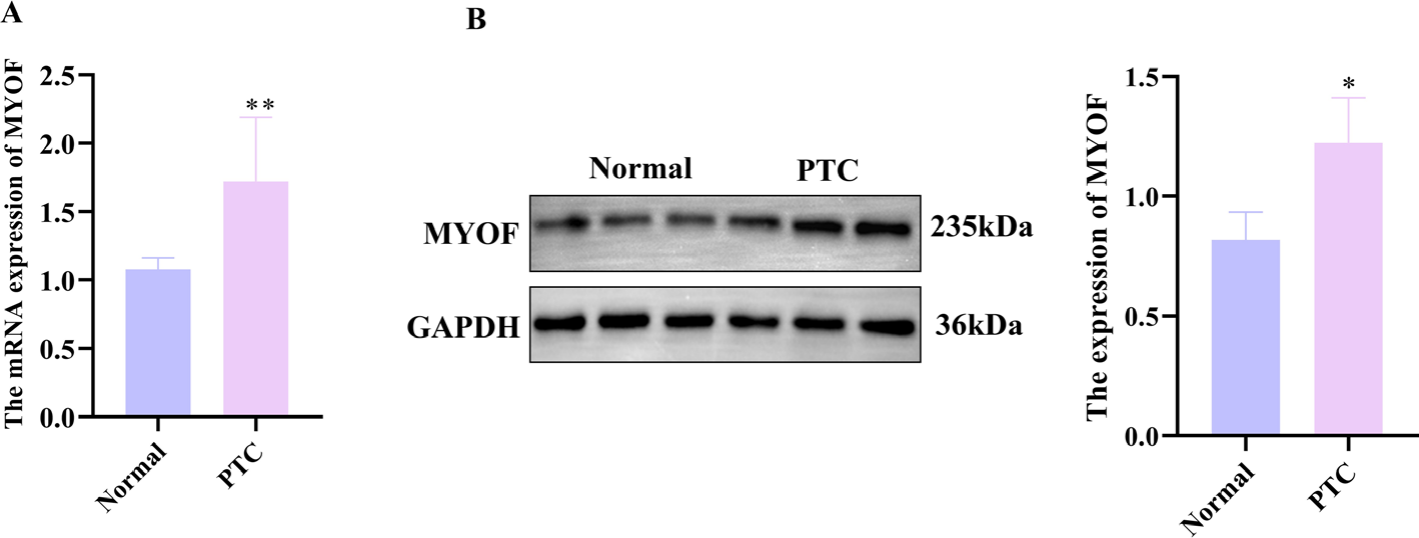
MYOF expression in PTC and adjacent noncancerous tissues. (A) qRT‐PCR showed significantly elevated MYOF mRNA levels in 3 PTC tumor samples relative to three paired adjacent noncancerous samples (**p < 0.01 vs. controls). (B) Western blot confirmed upregulated MYOF protein expression in the same set of three paired tumor and noncancerous samples (*p < 0.05 vs. controls).

To identify MYOF expression signatures under pathological conditions, we first collected paired samples comprising three sets of adjacent normal thyroid tissues and PTC tissues. MYOF expression in normal and PTC tissues was quantified via western blotting and qRT‐PCR. Analyses revealed significant upregulation of both MYOF protein and mRNA in tumor tissues versus patient‐matched controls (Figure 1A,B; *p* < 0.01, *p* < 0.05).

### 3.2 Investigating the Effects of MYOF Knockdown on Cellular Functions

**FIGURE 2:**
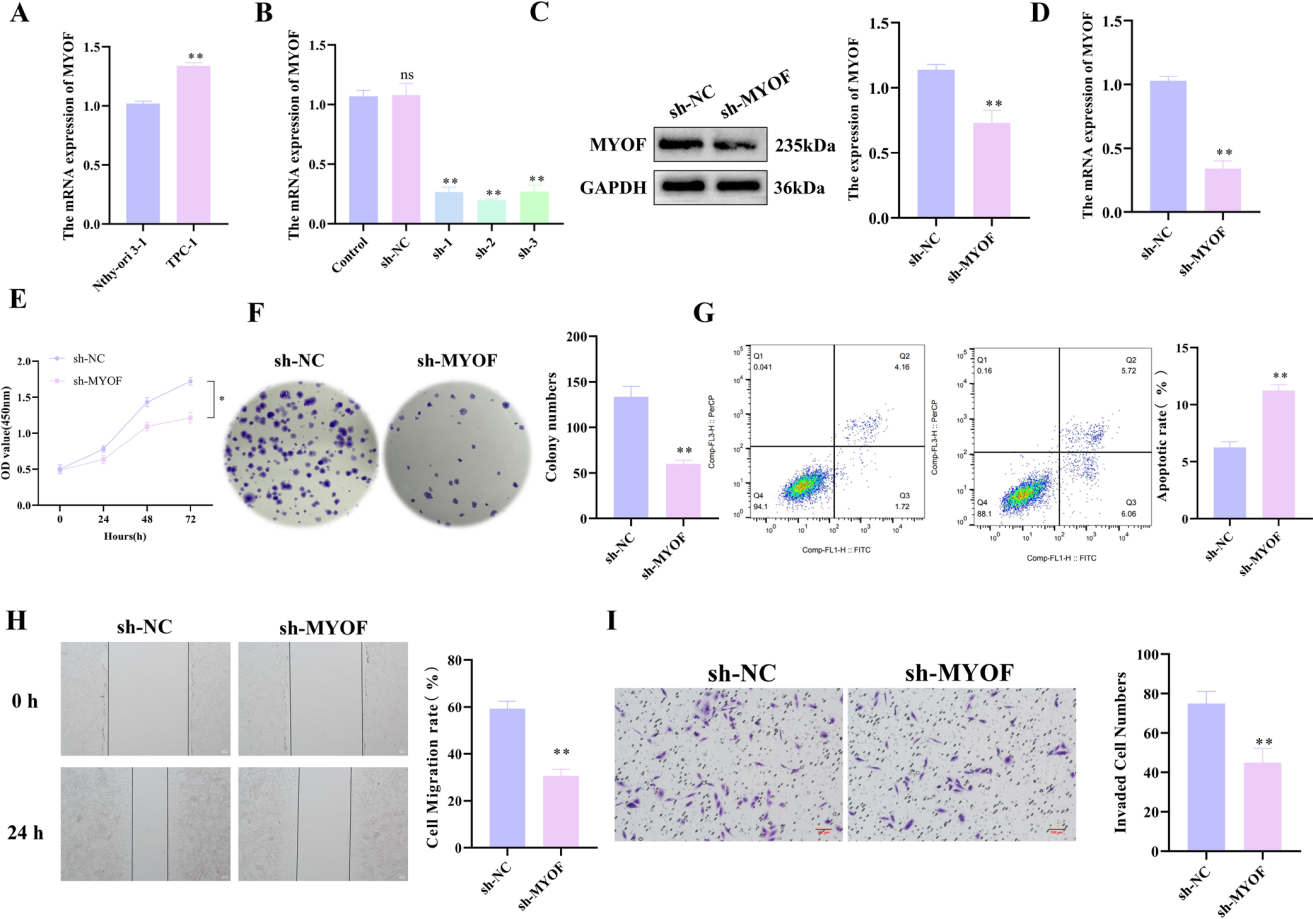
Investigating the effects of MYOF knockdown on TPC‐1 cells. (A) Comparative MYOF mRNA levels in TPC‐1 cells by qRT‐PCR. **p < 0.01 versus Nthy‐ori 3–1 group. (B) The relative expression level of MYOF after transfection with small interfering RNA was quantified using qRT‐PCR; **p < 0.01, versus si‐NC group. (C,D) MYOF protein and mRNA levels after knockdown by western blot and qRT‐PCR (*n* = 3 biological replicates), **p < 0.01 versus sh‐NC group. (E) Effect of MYOF knockdown on cell proliferation assessed using CCK‐8 assay (0, 24, 48, and 72 h) *p < 0.05 versus sh‐NC group. (F) Colony‐forming potential was measured using a colony formation assay. **p < 0.01 versus sh‐NC group. (G) Apoptotic cells were quantified using flow cytometry. **p < 0.01 versus sh‐NC group. (H) Wound healing assays quantified cell migration. Scale = 100 μm. **p < 0.01 versus sh‐NC group. (I) Cell invasion assessed using transwell chambers. Scale = 100 μm. **p < 0.01 versus sh‐NC group.

Comparative qRT‐PCR analysis of thyroid cell models revealed pronounced MYOF mRNA overexpression in neoplastic TPC‐1 cells compared with noncancerous Nthy‐ori 3–1 epithelial cells (Figure 2A, *p* < 0.01). We then established a stable MYOF‐knockdown model in TPC‐1 cells using lentiviral shRNA transduction. Further, qRT‐PCR screening identified sh‐MYOF‐2 as the most effective construct for MYOF knockdown, which was consequently used for subsequent experiments (Figure 2B, *p* < 0.01). Both western blot and qRT‐PCR confirmed successful MYOF knockdown, demonstrating significantly reduced MYOF protein and mRNA levels in sh‐MYOF TPC‐1 cells compared with the sh‐NC group (Figure 2C,D, *p* < 0.01). Functional assays demonstrated that MYOF depletion significantly impaired TPC‐1 cell biology. CCK‐8 assays indicated that the proliferative capacity of the sh‐MYOF group was significantly reduced over time compared with the sh‐NC group (Figure 2E, *p* < 0.05). This was corroborated by colony formation assays, where sh‐MYOF cells demonstrated markedly lower colony‐forming ability (Figure 2F, *p* < 0.01). Conversely, flow cytometry detected significantly increased apoptosis rates in MYOF‐knockdown cells (Figure 2G, *p* < 0.01). Furthermore, wound healing assays demonstrated decreased cell migration (Figure 2H, *p* < 0.01), and transwell invasion assays confirmed significantly reduced invasive capability upon MYOF knockdown (Figure 2I, *p* < 0.01).

**FIGURE 3:**
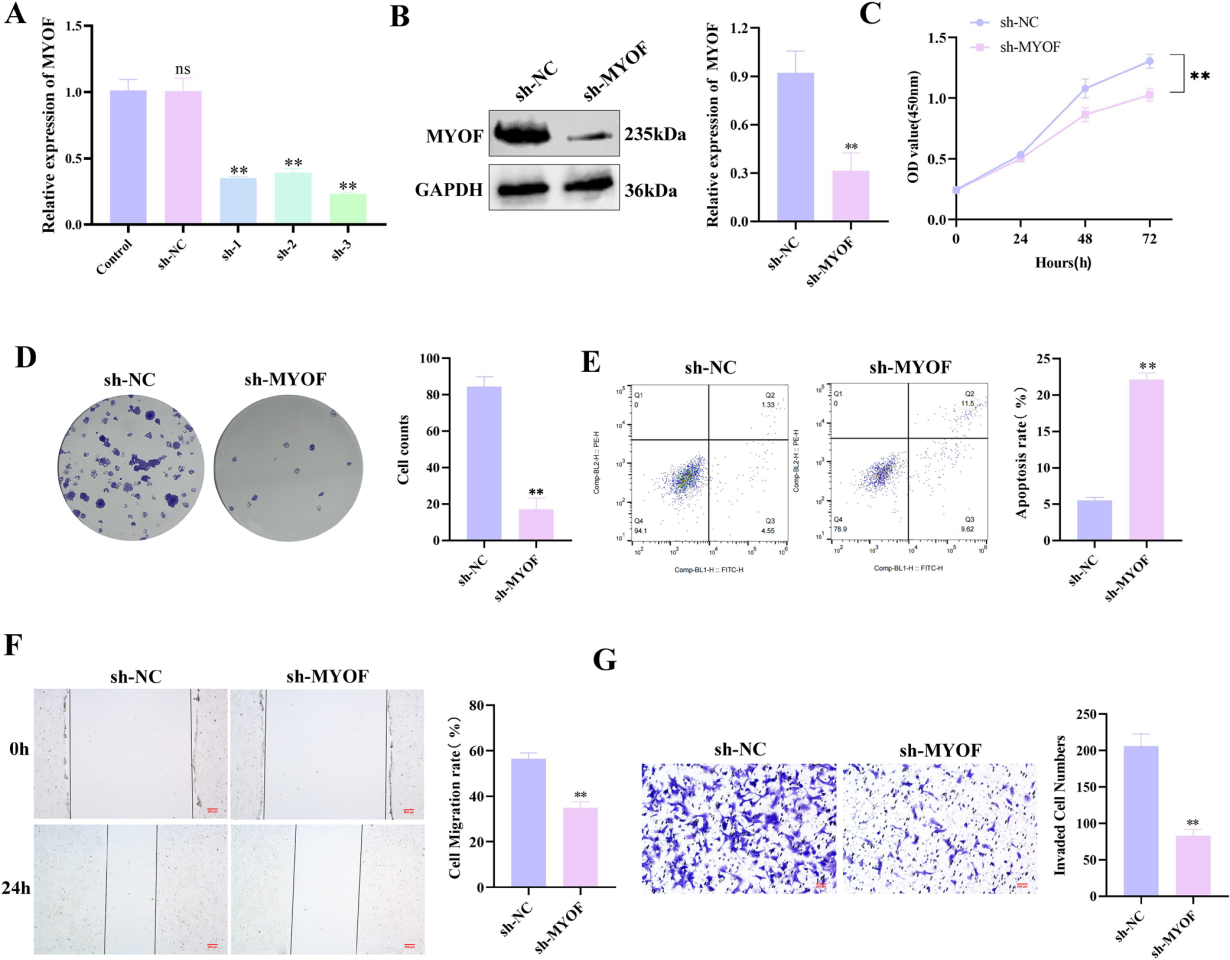
Investigating the effects of MYOF knockdown on KTC‐1 cells. (A) The relative expression level of MYOF after transfection with small interfering RNA was quantified using qRT‐PCR; **p < 0.01, versus si‐NC group. (B) MYOF protein levels after knockdown by western blot (*n* = 3 biological replicates), **p < 0.01 versus sh‐NC group. (C) Effect of MYOF knockdown on cell proliferation assessed using CCK‐8 assay (0, 24, 48, and 72 h) **p < 0.01 versus sh‐NC group. (D) Colony‐forming potential was measured using a colony formation assay. **p < 0.01 versus sh‐NC group. (E) Apoptotic cells were quantified using flow cytometry. **p < 0.01 versus sh‐NC group. (F) Wound healing assays quantified cell migration. **p < 0.01 versus sh‐NC group. Scale = 200 μm. (G) Cell invasion assessed using transwell chambers. Scale = 200 μm. **p < 0.01 versus sh‐NC group.

Moreover, to further validate the tumor‐suppressive role of MYOF in a more comprehensive manner, MYOF knockdown in the KTC‐1 cell line was performed (Figure 3A, *p* < 0.01), followed by a series of functional assays. Western blot analysis confirmed efficient knockdown of MYOF at the protein level (Figure 3B, *p* < 0.01). sh‐MYOF markedly suppressed cell proliferation and colony formation, while significantly enhancing apoptosis in KTC‐1 cells (Figure 3C–E, *p* < 0.01). Consistently, MYOF knockdown also attenuated the migratory and invasive capacities of TPC‐1 cells (Figure 3F,G, *p* < 0.01). Collectively, these functional data demonstrate that silencing MYOF significantly affects key cellular processes in PTC cells.

### 3.3 MYOF Depletion Induces Mitochondrial Dysfunction and Mitophagy

**FIGURE 4:**
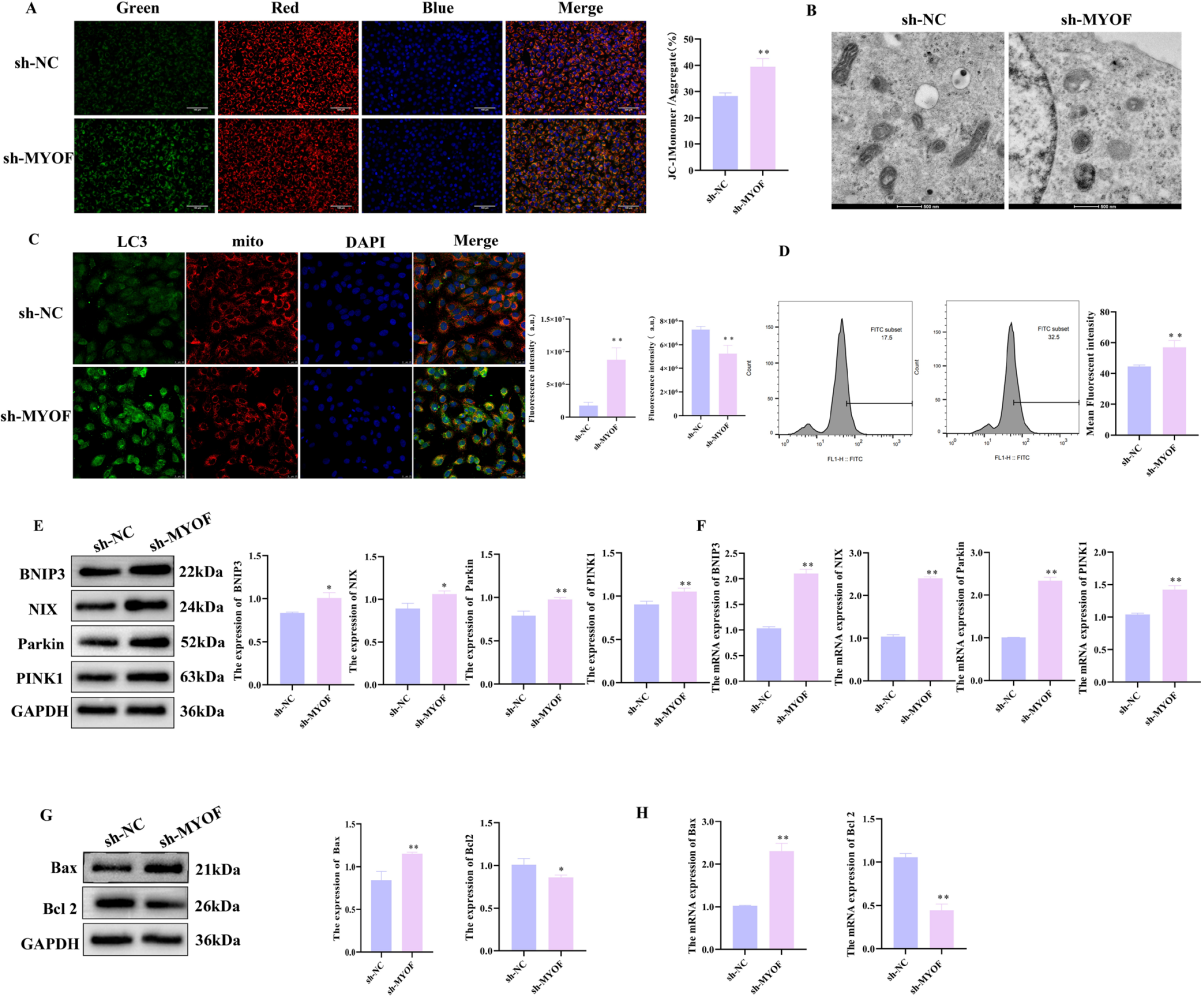
MYOF depletion induces mitochondrial dysfunction and mitophagy. (A) Mitochondrial polarization assessed via JC‐1 (Red: J‐aggregates; Green: JC‐1 monomer; blue: DAPI. Scale = 100 μm, **p < 0.01 vs. sh‐NC group). (B) TEM analysis of mitochondrial ultrastructure, **p < 0.01 versus sh‐NC group. Scale = 500 nm. (C) Confocal microscopy detection of mitochondrial–autophagosomal colocalization (Mitochondria: MitoTracker Red; Autophagosomes: LC3‐GFP. Scale = 25 μm, **p < 0.01 vs. sh‐NC group). (D) Intracellular ROS detection via flow cytometry, **p < 0.01 versus sh‐NC group. (E,F) Expression of mitophagy regulators (PINK1, Parkin, NIX, and BNIP3) at protein and transcript levels (*n* = 3 biological replicates), **p < 0.01 versus sh‐NC group. (G,H) Quantification of Bcl‐2 and Bax proteins (*n* = 3 biological replicates), **p < 0.01 versus sh‐NC group.

To investigate the association between MYOF and mitochondrial homeostasis, we first assessed mitochondrial membrane potential (ΔΨm) using JC‐1 immunofluorescence. Relative to the sh‐NC control group, the sh‐MYOF group demonstrated increased green fluorescence intensity with decreased red fluorescence, predominantly localized in the cytoplasm (Figure 4A, *p* < 0.01), indicating mitochondrial membrane potential (ΔΨm) depolarization. This indicates that MYOF may regulate cellular energy metabolism. TEM revealed that MYOF knockdown significantly increased autophagic vacuoles that contain mitochondrial debris while inducing ultrastructural alterations in mitochondria (Figure 4B). MitoTracker Red/LC3‐GFP colocalization analysis further quantified mitophagic flux. Further, sh‐MYOF cells demonstrated significantly enhanced colocalization (Figure 4C, *p* < 0.01), indicating increased mitophagy. Consistent with mitochondrial stress, flow cytometric quantification demonstrated increased ROS generation in sh‐MYOF cells (Figure 4D, *p* < 0.01). Molecular analysis confirmed these results. Western blot and qRT‐PCR revealed upregulation of mitophagy‐related proteins (PINK1, Parkin, NIX, and BNIP3) and the proapoptotic factor Bax at both protein and transcript levels in sh‐MYOF cells (Figure 4E,F, *p* < 0.01). Conversely, anti‐apoptotic Bcl‐2 expression was suppressed (Figure 4G,H, *p* < 0.01). Collectively, these data demonstrate that MYOF depletion disrupts mitochondrial integrity, activates PINK1‐Parkin‐mediated mitophagy, and promotes mitochondrial‐dependent apoptosis.

**FIGURE 5:**
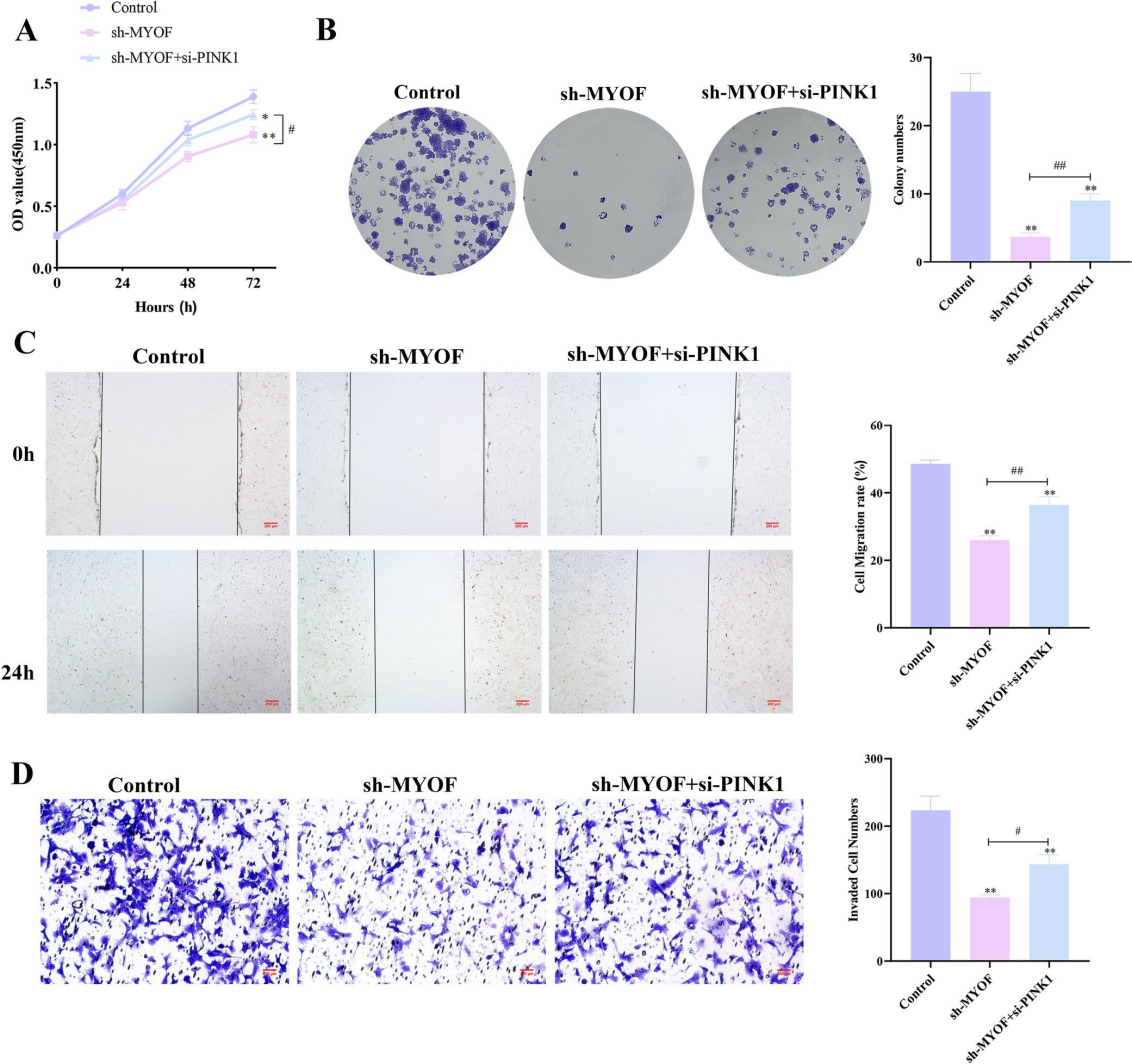
PINK1 silencing reverses the inhibitory effects of MYOF knockdown on cell proliferation, migration, and invasion. (A) Effect of PINK1 knockdown on cell proliferation assessed using CCK‐8 assay (0, 24, 48, and 72 h) *p < 0.05, **p < 0.01 versus control group, ^#^ *p* < 0.05 versus sh‐MYOF group. (B) Colony‐forming potential was measured using a colony formation assay. **p < 0.01 versus control group, ^##^ *p* < 0.01 versus sh‐MYOF group. (C) Wound healing assays quantified cell migration. **p < 0.01 versus control group, ^##^ *p* < 0.01 versus sh‐MYOF group. (D) Cell invasion assessed using transwell chambers. Scale = 200 μm. **p < 0.01 versus control group, ^#^ *p* < 0.05 versus sh‐MYOF group.

**FIGURE 6:**
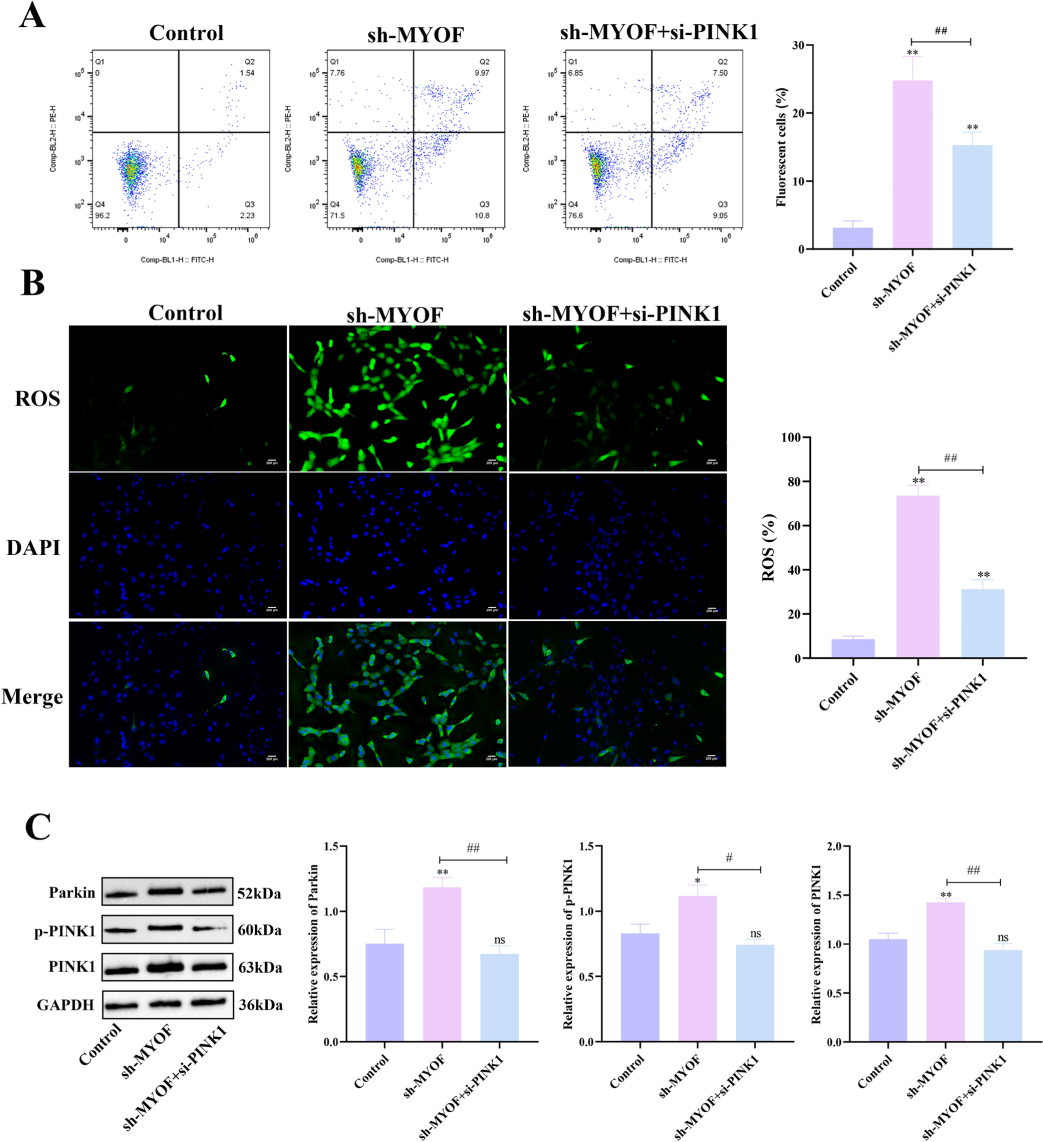
Knockdown of MYOF induces apoptosis and oxidative stress in a PINK1/Parkin‐dependent manner. (A) Apoptotic cells were quantified using flow cytometry. **p < 0.01 versus control group, ^##^ *p* < 0.01 versus sh‐MYOF group. (B) ROS detection was performed by immunofluorescence (Scale = 200 μm). **p < 0.01 versus control group, ^##^ *p* < 0.01 versus sh‐MYOF group. (C) Western blot analysis of Parkin and p‐PINK1 protein expression. Ns: Not significant, **p < 0.01 versus control group, ^#^ *p* < 0.05, ^##^ *p* < 0.01 versus sh‐MYOF group (*n* = 3 biological replicates).

Based on these findings, to further elucidate the role of the PINK1‐PARKIN pathway in MYOF‐mediated mitophagy, PINK1 was additionally silenced in the sh‐MYOF cell model via siRNA transfection. A series of functional assays demonstrated that PINK1 knockdown abrogated the inhibitory effect of sh‐MYOF on TPC‐1 cell proliferation (Figure 5A,B, *p* < 0.05). Furthermore, wound healing and transwell invasion assays validated this observation, confirming that si‐PINK1 reversed the suppressive effects of sh‐MYOF on the migratory and invasive capacities of TPC‐1 cells (Figure 5C,D, *p* < 0.05, *p* < 0.01). Meanwhile, flow cytometry analysis revealed a significant reduction in the apoptotic rate of TPC‐1 cells in the sh‐MYOF+si‐PINK1 group compared with the sh‐MYOF group (Figure 6A, *p* < 0.01); additionally, PINK1 silencing via siRNA led to a marked decrease in ROS fluorescence intensity in TPC‐1 cells (Figure 6B, *p* < 0.01). Moreover, sh‐MYOF upregulated the phosphorylation level of PINK1, whereas si‐PINK1 transfection significantly attenuated its phosphorylated expression (Figure 6C, *p* < 0.01). Taken together, these results further clarify the functional importance of the PINK1‐PARKIN pathway and the critical role of PINK1 phosphorylation in the regulation of MYOF‐mediated mitophagy.

### 3.4 MYOF Depletion Suppresses Tumor Growth via PINK1/Parkin‐Mediated Mitophagy Activation

**FIGURE 7:**
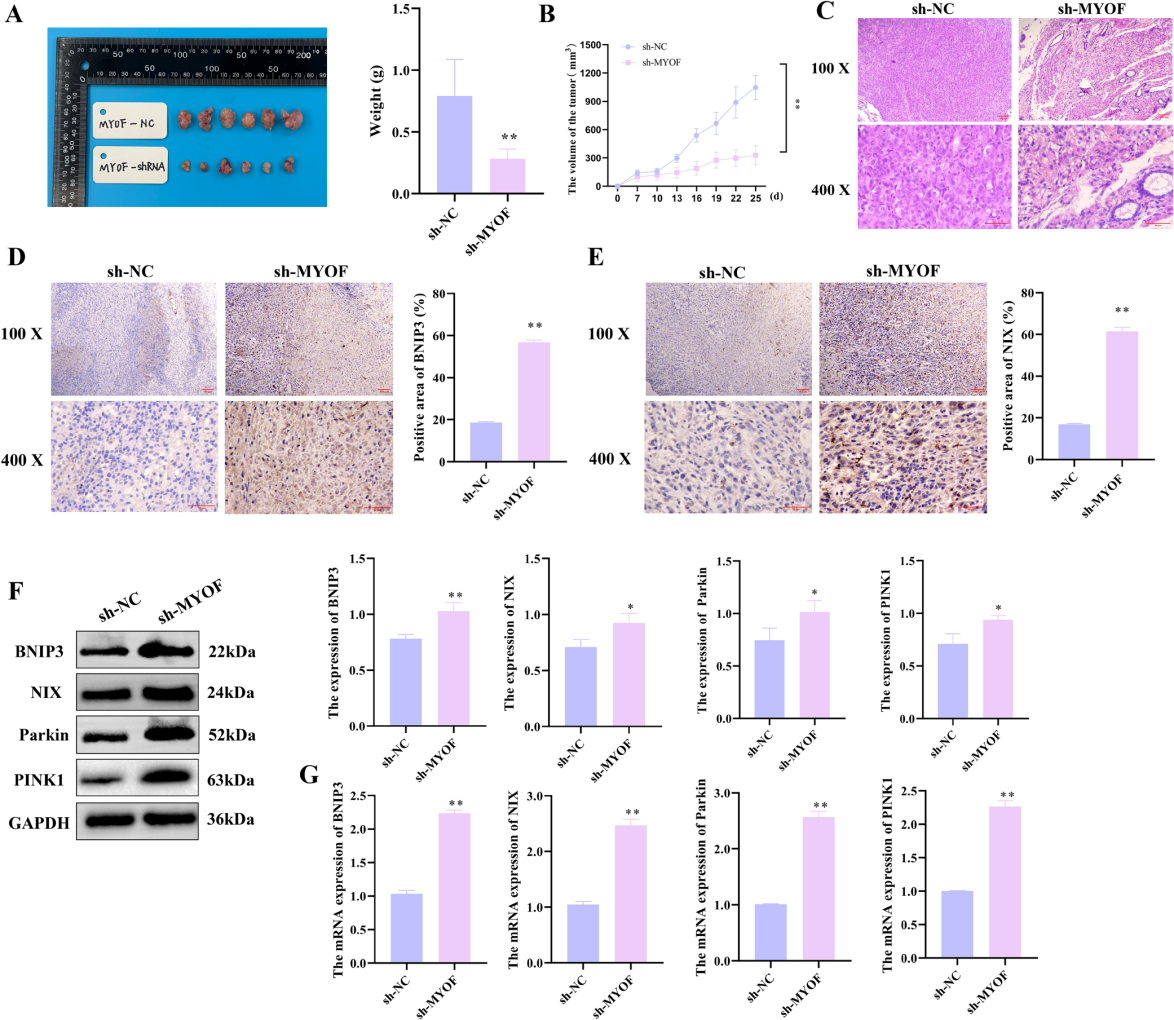
MYOF depletion suppresses tumor growth via PINK1/Parkin‐mediated mitophagy activation. (A,B) In vivo tumor growth kinetics in subcutaneous xenografts (*n* = 6 in each group),**p < 0.01 versus sh‐NC group. (C) HE staining reveals disorganized architecture and multifocal necrosis in sh‐MYOF tumors, with the upper and lower panels representing images at ×100 magnification (Scale = 100 μm) and ×400 magnification (Scale = 50 μm), respectively. (D,E) Immunohistochemical upregulation of mitophagy receptors: BNIP3 and NIX in the sh‐MYOF group, with the upper and lower panels representing images at ×100 magnification (Scale = 100 μm) and ×400 magnification (Scale = 50 μm), respectively. (F) Western blot analysis was conducted to assess the expression of mitophagy pathway‐related proteins (*n* = 3 biological replicates). (G) qRT‐PCR was performed to assess the expression of PINK1, Parkin, BNIP3, and NIX in sh‐MYOF tumors,**p < 0.01 versus sh‐NC group.

In vivo investigation revealed that MYOF knockdown conferred significant tumor‐suppressive effects in subcutaneous xenograft models, as evidenced by longitudinal growth kinetics analysis, demonstrating potent inhibition relative to sh‐NC control (Figure 7A,B, *p* < 0.01). Histopathological analysis revealed disorganized cellular architecture with multifocal necrosis in sh‐MYOF tumors (Figure 7C). Immunohistochemical staining demonstrated marked upregulation of mitophagy receptors BNIP3 and NIX (Figure 7D,E). Western blot and qRT‐PCR analyses further validated the activation of the PINK1/Parkin‐BNIP3/NIX axis with coordinated increases in PINK1, Parkin, BNIP3, and NIX expression (Figure 7F,G). Collectively, these results indicate that MYOF depletion suppresses tumor growth by potentiating PINK1/Parkin‐mediated mitophagy, leading to the inhibition of proliferative and metastatic potential. This work provides experimental validation for targeting mitophagy as a novel therapeutic paradigm in anticancer regimens.

### 3.5 MYOF Expression Is Associated With Lymph Node Metastasis (N Stage) in TC

Compared with normal thyroid tissues, MYOF expression was significantly higher in THCA tumor tissues (*p* = 0.0076, Figure S1A). Analysis of clinicopathological staging revealed that MYOF expression was significantly higher in N1 than in N0 stage (*p* = 0.01, Figure S1B). However, no significant differences in MYOF expression were observed among different T stages or M stages (*p* > 0.05, Figure S1C,D). With advancing pathological grade, MYOF expression exhibited a gradually increasing trend; however, this trend did not reach statistical significance (*p* > 0.05, Figure S1E). Univariate Cox regression analysis showed that MYOF was not a significant prognostic factor for overall survival (HR = 0.9988, 95% CI: 0.9872–1.0100). Consistently, log‐rank analysis based on median expression stratification revealed no significant survival difference between high‐ and low‐MYOF expression groups (*p* = 0.75; Figure S1F).

## 4 Discussion

PTC, the predominant thyroid malignancy, demonstrates a sustained annual incidence increase of 3%–4% (exceeding 4% in younger cohorts), requiring urgent clinical prioritization [17]. Crucially, PTC progression is caused by dysregulated organelle homeostasis—particularly mitochondrial dysfunction and endoplasmic reticulum stress—which collectively fuel tumor aggressiveness [18].

MYOF is a multi‐transmembrane protein belonging to the Ferlin family that regulates membrane repair, vesicle trafficking, and cell migration, with established oncogenic roles across solid tumors: TRIM8‐mediated K48‐linked ubiquitination targets MYOF for proteasomal degradation to suppress lung cancer metastasis [19]. MYOF upregulation is a prognostic biomarker in ovarian cancer [20], and pharmacological inhibition of its C2D domain by WJ460 attenuates breast cancer invasion and metastasis [21, 22], whereas MYOF triggers mitophagy and ferroptosis in pancreatic cancer [23]. This study bridges a critical knowledge gap by establishing MYOF as a pivotal oncogenic driver in PTC through mitophagy suppression: clinical specimens revealed significant MYOF upregulation in PTC tissues, supporting its diagnostic/prognostic use; stable lentiviral shRNA‐mediated knockdown suppressed malignant phenotypes—including proliferation, migration, invasion, and colony formation—while inducing proapoptotic effects. Mechanistically, targeted MYOF silencing inhibits PTC progression by counteracting pathological hyperproliferation and apoptosis resistance, thereby providing the first evidence of MYOF's tumor‐promoting function in thyroid malignancies.

Extensive research has revealed mitophagy dysregulation as a critical oncogenic driver in malignancies, evidenced by mtDNA, transfer RNA (tRNA) mutations that impair respiratory complexes to suppress breast cancer [24], oxidative damage‐driven colorectal carcinogenesis associated with increased mitochondrial protein carbonylation [25], and PINK1‐BNIP3 axis‐mediated tumor metastasis via precise Parkin signaling regulation [26, 27]. However, significant controversies persist, as pulmonary fibrosis models demonstrate PINK1 deficiency activates BNIP3‐mediated mitophagy for tissue protection, whereas Parkinson's pathology demonstrates disrupted PINK1‐BNIP3 crosstalk [28, 29]. Within this context, we identify MYOF as a novel master regulator of mitophagy in PTC. Silencing this membrane repair protein compromises mitochondrial outer membrane integrity, thereby inducing ΔΨ collapse that triggers ROS accumulation and irreversible ultrastructural damage. Initial PINK1/Parkin‐mediated mitophagy provides cytoprotection; however, sustained injury causes autophagic flux overload that exceeds lysosomal degradation capacity, ultimately submitting cells to apoptosis through Bax/Bcl‐2 imbalance and caspase‐3 cleavage. This establishes a MYOF‐centric regulatory paradigm distinct from BNIP3‐dominated mechanisms in other pathologies, which provides the first evidence that MYOF deficiency leads to PTC progression through sequential mitochondrial events: ΔΨ destabilization resulting in PINK1‐Parkin axis hyperactivation, which initiates compensatory mitophagic flux.

Autophagy, an evolutionarily conserved self‐clearance mechanism, facilitates the sequestration and lysosomal degradation of dysfunctional cellular components to maintain intracellular physiological homeostasis, thereby exerting a cytoprotective effect in stressed cells [30]. Notably, this process plays a dual role in the initiation, progression, and chemoresistance of cancer [31]. In this study, MYOF knockdown‐induced disturbance of mitochondrial membrane potential was accompanied by elevated ROS levels, indicating that MYOF deficiency elicits mitochondrial dysfunction and exacerbates oxidative stress in cancer cells. Our findings are consistent with previous studies. For instance, piperlongamine has been shown to induce autophagy and suppress tumor growth in thyroid cancer cells by triggering ROS production and the subsequent inhibition of the Akt/mTOR signaling pathway [32]. Similarly, KIF23 modulates mitophagy via the Wnt/β‐catenin axis and thereby regulates the migratory capacity of PTC cells [33]. Building on these findings, our study further extends this research scope by verifying that MYOF knockdown activates PINK1/Parkin‐mediated mitophagy to facilitate the clearance of damaged mitochondria, while the initial ROS surge may further amplify autophagic flux through feed‐forward signaling, ultimately driving PTC cells toward apoptotic cell death.

By establishing a subcutaneous xenograft model in nude mice [34], our in vivo investigations confirmed that MYOF silencing significantly inhibited tumor growth in TPC‐1‐derived transplants, with histopathological examination revealing multifocal necrosis and disrupted cellular architecture. Molecularly, MYOF deficiency concurrently activated dual mitophagic pathways through coordinated upregulation of key regulators PINK1/Parkin and BNIP3/NIX. This finding addresses the prevailing research gap wherein current studies focus predominantly on isolated functions of either the PINK1‐Parkin axis (e.g., pancreatic cancer) or BNIP3 (e.g., breast cancer), thereby providing novel therapeutic insights for PTC management [35, 36]. Importantly, mitophagy activation has been documented to enhance the sensitivity of cancer cells to chemotherapy and targeted therapeutics across various malignancies [37]. In our experimental model, the knockdown of MYOF induced mitophagy, which, in conjunction with elevated ROS levels, may collectively compromise the antioxidant defense system of PTC cells, thereby potentially increasing their susceptibility to clinical first‐line therapies. These findings provide a novel mechanistic basis for targeting MYOF in PTC, as its inhibition not only directly suppresses tumorigenesis but may also reverse therapeutic resistance through the activation of autophagic signaling pathways. Collectively, our integrated in vitro and in vivo evidence indicates that MYOF represses mitophagy through negative regulation of the PINK1‐Parkin/BNIP3‐NIX signaling nexus, with its targeted inhibition eliciting autophagy‐dependent tumor suppression. These results indicate novel pathogenic mechanisms in PTC progression and propose a promising therapeutic strategy.

Although tumor metastasis and clinical prognosis represent core issues of clinical translational relevance, our TCGA‐based bioinformatic analysis revealed that elevated MYOF expression is significantly correlated with lymph node metastasis (N stage) in TC. This clinical observation is consistent with our in vitro and in vivo findings, which validate the regulatory function of MYOF in facilitating PTC cell migration and invasion. Nevertheless, no significant correlations were observed between MYOF expression and T stage, M stage, or pathological grade. Meanwhile, survival analysis indicated that MYOF could not serve as an independent prognostic biomarker for overall survival in THCA. While these results seem to restrict the potential of MYOF as a single prognostic indicator, they do not compromise the core significance of the present study. Instead, our data support that MYOF exerts a specialized function in promoting local lymphatic spread, rather than driving distant metastasis or affecting long‐term patient survival. Lymph node metastasis is a critical early event in TC progression, which is tightly associated with tumor recurrence and clinical therapeutic decision‐making [38]. Accordingly, our discovery that MYOF contributes to lymphatic metastasis identifies a promising molecular target for blocking the early metastatic cascade in TC. Such targeted interventions confer important clinical value, even if MYOF shows no obvious impact on overall survival. Collectively, these results emphasize the necessity of context‐specific interpretation of cancer biomarkers, and suggest that therapeutic targeting of MYOF may offer clinical benefits for TC patients with high risks of lymph node metastasis.

This study delineates MYOF's pivotal role in regulating PTC mitophagy through TPC‐1 models; however, several limitations of the present study should be acknowledged. First, the sample size of clinical specimens was relatively small. Second, this work lacks MYOF overexpression experiments, and it remains unconfirmed whether MYOF overexpression directly suppresses mitophagy. In addition, MYOF knockdown induces mitochondrial dysfunction, which may indirectly trigger mitophagy as a secondary cellular response. Furthermore, although our study revealed a correlative relationship, concrete evidence for direct molecular interaction is still insufficient. The precise regulatory crosstalk between MYOF and the PINK1‐Parkin/BNIP3‐NIX signaling axis also needs to be further explored. In future research, we will conduct MYOF overexpression assays to verify its biological function in mitophagy. Meanwhile, we will perform comprehensive characterization of MYOF expression and functional landscapes across genetically distinct PTC subtypes, notably BRAF and RAS‐mutant variants, followed by mechanistic dissection using integrated strategies spanning protein interactome profiling and structure‐guided mutagenesis.

## 5 Conclusion

Collectively, this study demonstrates that MYOF drives PTC progression by suppressing mitophagy, whereas targeted MYOF knockdown activates mitophagic flux, thereby inhibiting oncogenic properties, including proliferation and migration, ultimately attenuating PTC pathogenesis.

## Ethics Statement

The research protocol received official approval from the Ethics Committee of First Affiliated Hospital of Harbin Medical University (Authorization Code: IRB‐AF/SC‐08/07.0), with written informed consent documents secured from every enrolled participant. The animal experiments were approved by the Ethics Committee of Experimental Animal Welfare and Ethics Committee of Jilin Bairen Medical Laboratory Co. LTD (BR.No. 20250306001).

## Conflicts of Interest

The authors declare no conflicts of interest.

## Supporting information


**Figure S1:** Clinical analysis of MYOF expression in the TCGA‐THCA cohort. (A) Expression levels of MYOF (TPM) in tumor versus normal thyroid tissues. The *y*‐axis represents the transcript abundance of MYOF, quantified as transcripts per million (TPM). (B–E) Expression levels of MYOF across different (B) N stages, (C) T stages, (D) M stages, and (E) different pathologic stages. (F) Kaplan–Meier survival curves comparing overall survival between patients with high and low MYOF expression in the TCGA‐THCA dataset. The *y*‐axis indicates survival probability, and the *x*‐axis represents follow‐up time in days.

## Data Availability

Data sharing not applicable to this article as no datasets were generated or analysed during the current study.
