# Supplementary figures and images for: The Regulatory Role and Mechanism of Myoferlin in Mitophagy During Papillary Thyroid Carcinogenesis
Source: Kaohsiung J Med Sci. 2026 Jun 10:e70245. Online ahead of print. doi: 10.1002/kjm2.70245 (PMC13399744; doi:10.1002/kjm2.70245)

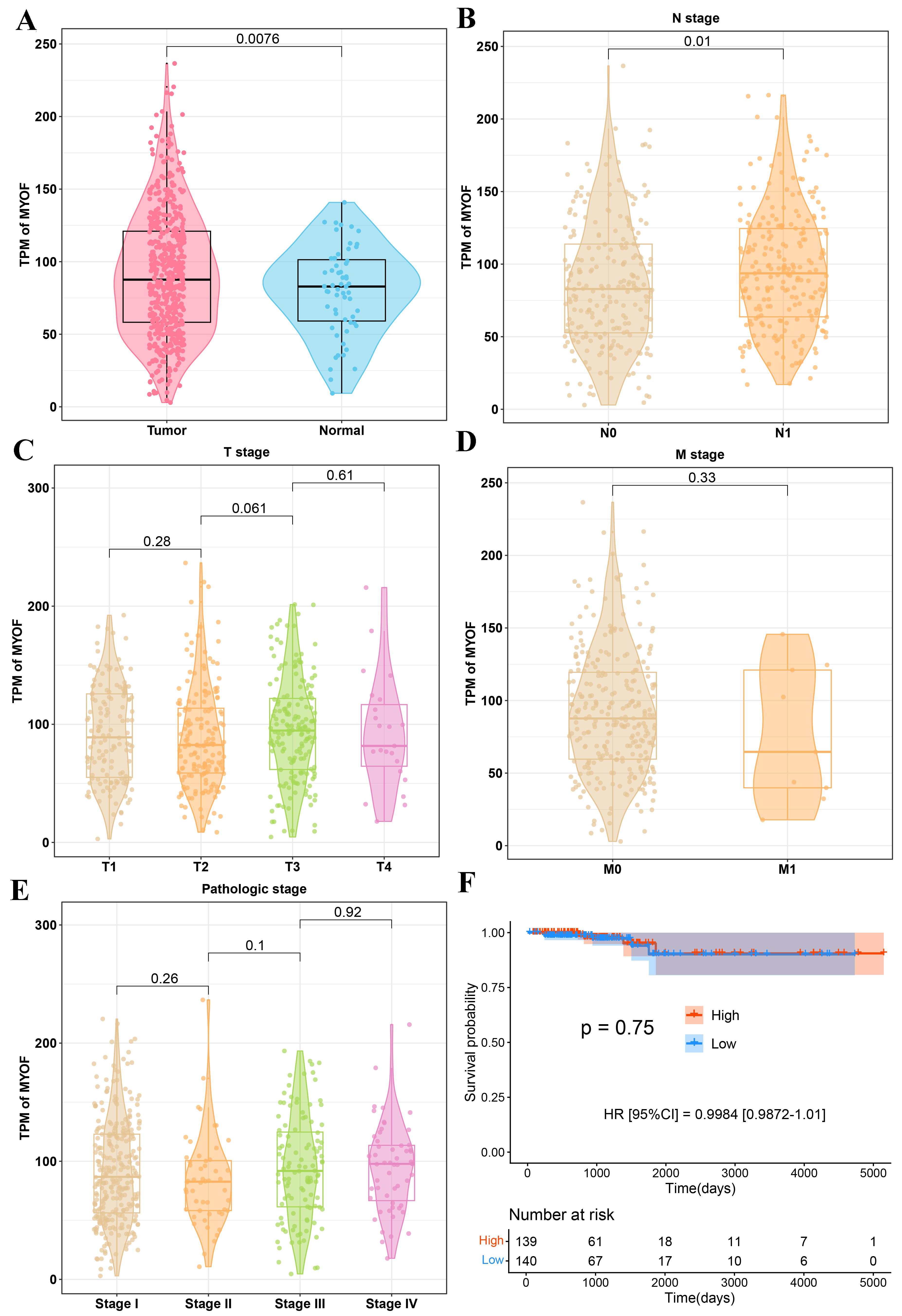

Supplement: Supplementary file 1 — Figure S1: Clinical analysis of MYOF expression in the TCGA‐THCA cohort. (A) Expression levels of MYOF (TPM) in tumor versus normal thyroid tissues. The y‐axis represents the transcript abundance of MYOF, quantified as transcripts per million (TPM). (B–E) Expression levels of MYOF across different (B) N stages, (C) T stages, (D) M stages, and (E) different pathologic stages. (F) Kaplan–Meier survival curves comparing overall survival between patients with high and low MYOF expression in the TCGA‐THCA dataset. The y‐axis indicates survival probability, and the x‐axis represents follow‐up time in days. [file KJM2-9999-e70245-s001.jpg]
